# Biomass-Derived Carbon Aerogels for ORR/OER Bifunctional Oxygen Electrodes

**DOI:** 10.3390/nano13172397

**Published:** 2023-08-23

**Authors:** Yue Jiao, Ke Xu, Huining Xiao, Changtong Mei, Jian Li

**Affiliations:** 1 Co-Innovation Center of Efficient Processing and Utilization of Forest Resources—International Innovation Center for Forest Chemicals and Materials, Joint International Research Laboratory of Lignocellulosic Functional Materials, College of Materials Science and Engineering, Nanjing Forestry University, Nanjing 210037, China; xukekexu@njfu.edu.cn (K.X.); mei@njfu.edu.cn (C.M.); 2 Chemical Engineering Department, New Brunswick University, Fredericton, NB E3B 5A3, Canada; hxiao@unb.ca.com; 3 Material Science and Engineering College, Northeast Forestry University, Harbin 150040, China

**Keywords:** oxygen evolution reaction, oxygen reduction reaction, electrocatalyst, biomass, carbon aerogel

## Abstract

The oxygen reduction reaction (ORR) and oxygen evolution reaction (OER) are crucial electrochemical reactions that play vital roles in energy conversion and storage technologies, such as fuel cells and metal–air batteries. Typically, noble-metal-based catalysts are required to enhance the sluggish kinetics of the ORR and OER, but their high costs restrict their practical commercial applications. Thus, highly active and strong non-noble metal catalysts are essential to address the cost and durability challenge. Based on previous research, carbon-based catalysts may present the best alternatives to these precious metals in the future owing to their affordability, very large surface areas, and superior mechanical and electrical qualities. In particular, carbon aerogels prepared using biomass as the precursors are referred to as biomass-derived carbon aerogels. They have sparked broad attention and demonstrated remarkable performance in the energy conversion and storage sectors as they are ecologically beneficial, affordable, and have an abundance of precursors. Therefore, this review focuses on various nanostructured materials based on biomass-derived carbon aerogels as ORR/OER catalysts, including metal atoms, metal compounds, and alloys.

## 1. Introduction

In recent years, concerns regarding the energy crisis have emerged worldwide owing to the depletion of fossil fuels, environmental pollution, and global warming. Therefore, novel energy conversion devices and energy storage technologies are urgently required [1]. Rechargeable zinc–air batteries (ZABs) have been regarded as highly favorable candidates for next-generation energy storage devices owing to their numerous advantages, including high theoretical energy densities, plentiful resources, significant safety, and environmentally friendly properties. Consequently, ZABs are among the various energy conversion and storage systems that have gained considerable attention [2,3].

However, ZABs still encounter several significant challenges in terms of performance improvements and potential future commercialization. The greatest challenge here is the lack of an efficient air cathode, primarily owing to the slow kinetics involved in the oxygen reduction reaction (ORR) and oxygen evolution reaction (OER) during the charge–discharge process [4,5]. The performance of the catalyst is a crucial factor that determines the activity and selectivity of the ORR and OER. To date, noble metal catalysts based on Pt, Ru, and Ir are considered the most efficient catalysts with high catalytic activities. However, the large-scale industrial applications of these noble-metal-based catalysts are seriously limited by the high costs, scarcity, and separate selectivity of certain reactions [5]. Furthermore, realizing improvements in O_2_ reduction and evolution efficiencies is another major challenge for ZABs. Hence, it is necessary to develop stable and effective bifunctional electrocatalysts. For instance, Pt-based catalysts exhibit excellent ORR performance but poor OER activity owing to the formation of a stable oxide layer with low conductivity. Ru/Ir-based oxides exhibit excellent OER activity but low ORR activity [6,7]. This selectivity necessitates an additional manufacturing process for various catalysts for energy conversion devices. Consequently, efficient, durable, bifunctional, and inexpensive non-noble metal-based catalysts (NPMCs) are urgently required [8].

Non-noble metals, known as transition metals (e.g., Mn, Co, Ni, Fe, etc.), and their oxides, sulfides, nitrides, etc., have become popular substitutes for noble metal catalysts owing to their advantages such as low cost and abundant sources [9]. Additionally, electrocatalysts based on transition metals have a unique three-dimensional (3D) electronic configuration and demonstrate remarkable catalytic activity for the ORR and OER. Nevertheless, the wide-spread application of transition metal oxides in electrocatalysts is limited owing to their poor electrical conductivity [10]. Therefore, to enhance the catalytic activity, transition metal oxides are usually combined with carbon-based material supports such as graphene nanosheets, carbon nanotubes (CNT), and carbon fibers, which can provide a high electrical conductivity and large surface area for good electron transport and mass transportation [9,11,12]. However, some key challenges remain unaddressed: (i) the above-mentioned conventional carbon supports have a restricted surface area, which limits the exposure of active sites and mass transfer, and (ii) to realize the combination of non-noble metals and carbon support, it is usually necessary to deposit the catalyst particles either directly on the carbon support surface or to fix them by adsorption. However, these unstable physical interactions (non-intimate contact) between the catalyst particles and the substrate leads to poor stability [13,14]. Generally, 3D porous carbon aerogels can immobilize active particles more effectively than conventional carbon carriers by solving the above-mentioned problems to some extent and simultaneously facilitating catalytic reactions at the triple active phase boundary (air-electrolyte-catalyst) [13,15,16].

Close interactions between the active particles and carbon aerogel (CA) may result in a synergistic effect, significantly augmenting the active sites generated through electron transfer from the metal particles to the carbon layer, thus causing reductions in the local work function on the carbon surface. On the one hand, CAs exhibit superior advantages owing to their unique structures, such as high porosity, multi-dimensional charge transport paths, and superior electrical conductivity, which are highly conducive to enhancing both mass and electron transport. On the other hand, heteroatom doping is another approach for modifying CAs. Peroxide is mostly formed through the two-electron pathway from the pure carbon material with poor ORR activity [17]. By contrast, carbon materials with heteroatom doping of N, P, S, and B could stimulate the generation of OH^−^ through the four-electron pathway, thus adjusting the local charge distribution and electronic structure of the substrate carbon material. The ORR enhancement mechanism of heteroatom-doped carbon materials could be described as: heteroatoms having stronger electronegativity than carbon atoms, causing electron defect or structural disordering of adjacent carbon atoms and thus leading to the enhancement of oxygen adsorption on the carbon surface [7,18].

According to the types of precursors, CAs can be broadly classified into four categories: graphene CAs, polymer-based CAs, organic molecular-derived CAs, and biomass-based CAs [19]. Among them, the biomass-based CAs are primarily prepared by the carbonization of biomass aerogels with the direct achievement of a porous structure. Biomass is one of the most abundant renewable energies worldwide with great advantages such as low cost, versatile morphology, renewable and degradable characteristics, and plentiful carbon sources. Therefore, biomass aerogel carbonization is an economical and sustainable method for producing environmentally friendly porous CAs from abundant biomass resources. Plant cellulose, bacterial cellulose, and plants with 3D porous structures are the primary choices of biomass-based CA precursors.

This review aims to look into the potential of reliable and commercial approaches for ORR and OER electrocatalysis using biomass-based carbon aerogels. In recent years, several reviews have focused on CAs and electrocatalysis. Peles-Strahl et al., offered an overview of the recent developments and trends in aerogels-supported ORR catalysts, including different aerogel structures (CA and metal aerogels) [20]. Huang et al., presented advances in atomic-scale modulation and the nanostructure design of carbon-based bifunctional oxygen catalytic materials for metal–air batteries [21]. Shao et al., provided a comprehensive review on the customization of nanostructured carbon-based electrodes and carbon carrier design for ZAB. In this study, we focused on carbon-based catalysts, that is, CAs derived from biomass [22]. First, the preparation and characterization of biomass-based CAs are briefly described, focusing on the reaction mechanisms of the ORR and OER on air electrodes. Afterwards, the performance of various biomass-based carbon-aerogel-based materials developed by the ORR and OER in ZABs is summarized, highlighting the relationship between the active site structure and the electrocatalytic performance in oxygen electrocatalysis. Finally, the opportunities and challenges of biomass-based CAs for electrocatalytic applications are proposed.

## 2. Preparation and Modification of Biomass-Based CAs

CAs belong to a branch of aerogels. They are a relatively new type of porous carbon material obtained from the high-temperature pyrolysis of organic aerogels as precursors in an inert gas environment [23]. CAs are featured by the typical 3D nano-network porous structure of aerogels; however, they have several advantages, including high specific surface areas, high porosity, and large pore volumes [24]. Meanwhile, their preparation method is feasible and controllable, with tremendous potential for applications in the fields of catalyst supports [25], supercapacitors [23,26], and adsorption materials [27]. However, the industrial production and commercial application of CAs have not been realized yet, owing to the complex production process, long production cycle, small production scale, high cost of raw materials, and potential environmental pollution issues. Therefore, it is meaningful to continuously search for precursor substitutes that are abundant, inexpensive, and environmentally friendly.

**Figure 1:**
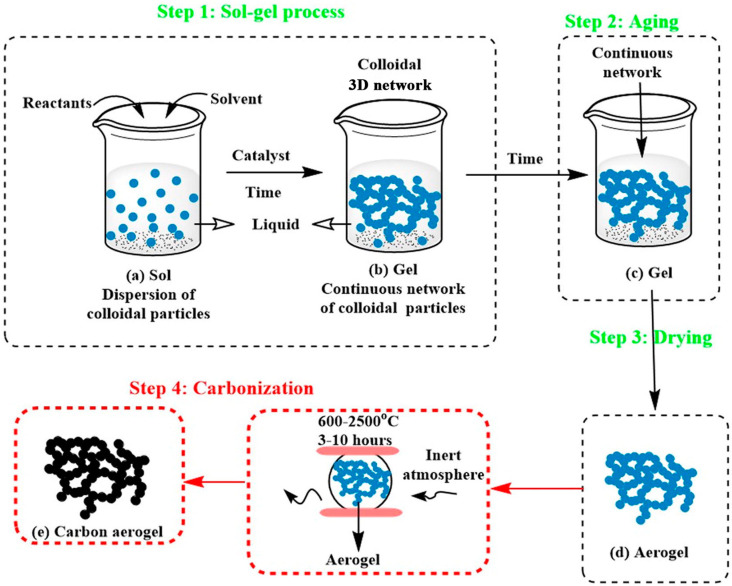
Schematic diagram for the preparation process of CA. Copyright 2016 by the Elsevier from [34].

Biomass-based materials are the most economical, non-polluting, and sustainable precursor for CA preparation as biomass resources are accessible, affordable, and renewable. Researchers have prepared CAs using a wide variety of biomass sources and its derivatives, such as chitosan, bacterial cellulose, wood, soy protein, and glucose [28,29,30,31,32,33]. The preparation process for biomass-based CAs comprises mainly three stages, i.e., sol-gel or dissolution regeneration, drying, and carbonization (as shown in Figure 1), and each stage has a certain influence on the final properties of the CAs. Supercritical drying or freeze drying is usually used to improve the pore parameters (specific surface area and porosity) of CAs and preserve the 3D porous network skeleton structure [34].

### 2.1. Sol-Gelation of Biomass-Based CAs

Most aerogels are prepared by wet chemical synthesis (sol-gel method), and their performance difference depends on various factors such as the precursor material type and preparation conditions [35]. For the sol-gel method, the sol usually refers to the dispersed solution of stable colloidal particles derived from the hydrolysis and polycondensation of the precursor, water, solvent, and catalyst. Notably, intimate contact among colloidal particles with the formation of a connected 3D network structure could be realized by the addition of chemical crosslinking agents or changes in the physical conditions. This process is key in determining whether the 3D porous structures of aerogels can be formed.

Therefore, one of the features of the sol-gel process is that the microstructure of the gel can be tuned by adjusting the reaction parameters, which, in turn, influence the properties of the final product, i.e., aerogel. The relevant parameters include precursor concentration, solvent type, pH, and temperature. Additionally, unique properties of aerogels can be realized by incorporating components other than precursors into the sol-gel process. For instance, Zhu et al., introduced polyamide-epichlorohydrin (PAE) into the system as an in situ crosslinking agent to enhance the mechanical stability of an aerogel. Meanwhile, methyltrimethoxysilane (MTMS) was chemical vapor deposited onto the aerogel to achieve hydrophobic modification. Another illustration is the preparation of an aerogel with magnetic or photocatalytic properties by adding magnetic or titanium dioxide nanoparticles [36]. Wang et al., successfully synthesized CoFe_2_O_4_ nanoparticles combined with N-doped reduced graphene oxide aerogels, which exhibit typical ferromagnetic behavior [37].

For biomass-based CAs, the precursors differ in the above-mentioned treatment processes. When using cellulose as the raw material, a cellulose micro-scale or nano-scale dispersion is usually obtained by acid hydrolysis, enzymatic hydrolysis, or mechanical treatment, in which the molecules in the sol are cross-linked via chemical bonds, hydrogen bonds, or van der Waals forces to produce a solid gel with a connected network structure. However, the sol-gel process is not required for biomass materials with a 3D network structure and bacterial cellulose because they are already in the crosslinking state. Wan et al., used straw-derived cellulose extracted as a raw material and prepared a new porous cellulose aerogel using the cellulose freeze-thaw regeneration method and freeze-drying method. By contrast, bacterial cellulose itself is in a gel state, and bacterial cellulose-based CAs can be directly prepared by freeze-drying and high-temperature carbonization [38]. Meng et al., prepared porous CAs using the following process: bacterial cellulose in liquid nitrogen was directly frozen (freeze-drying for 24 h), and high-temperature carbonization was conducted at 900 °C under nitrogen protection [39]. Li et al., also found that the fruits and rhizomes of some water-rich plants are in a sol-gel state, and CAs can be prepared by hydrothermal carbonization or direct carbonization while maintaining a 3D network structure [40].

### 2.2. Drying of Biomass-Based CAs

The drying treatment is a critical stage in the CA preparation process. At this stage, the solvent in the hydrogel is removed with the only residual of the solid network, namely aerogel. The drying process greatly impacts the ultimate structure and properties of the aerogel. The unsuitable drying method will cause massive shrinkage of the aerogel structure, destruction of the porous structure, and a change in the specific surface area. Currently, three drying methods are commonly used: ambient pressure drying, supercritical drying, and freeze drying.

(1) Ambient pressure drying

Ambient pressure drying is a drying process that converts a wet gel into a dry state by causing the evaporation of the gel solvent under atmospheric pressure. The current routine solvent evaporation may induce drastic changes in surface tension when forming the vapor–liquid interface. Due to the simultaneous presence of liquid/vapor phases and the large difference in surface tension, significant mechanical stresses are generated and consequently lead to the destruction of the pore structure under atmospheric pressure. The pore shrinkage of the aerogel prepared by ambient pressure drying is obvious. Furthermore, the shrinkage degree is also affected by the drying temperature and rate. When the temperature is higher, aerogel shrinkage is more obvious.

(2) Supercritical drying

Supercritical drying can solve some of the problems involved in conventional drying. To maintain the high porosity and superior properties of the wet gel under dry conditions, the wet gel is filled with a supercritical fluid (commonly liquid CO_2_). Then, liquid CO_2_ is directly transferred to the supercritical state without going through the vapor–liquid interface, which minimizes the mechanical stress generated by the solvent on the pore wall. Several researchers have used supercritical drying to prepare aerogels. This drying method avoids vapor–liquid phase conversion and surface tension in gel pores during solvent elimination, thus evading the pore collapse of the gel structure. The supercritical drying process can well avoid the damage of capillary pressure on the 3D structure of the gel. However, there are also some drawbacks as follows: (i) the equipment cost for supercritical drying is expensive, (ii) supercritical drying is usually performed under harsh conditions such as high temperature and pressure, leading to security risks, and (iii) the replacement process is time consuming, and the energy consumption is massive.

(3) Freeze drying

Compared to supercritical drying, freeze drying is a drying technique with advantages such as easy operation, low expenses, minimal energy consumption, and maintaining the 3D porous structure of the gel. The basic principle of freeze-drying is the sublimation of ice crystals. That is, the frozen water in the gel is directly sublimated to gas without melting into the liquid phase under high-vacuum conditions. The freeze-drying technique can also well preserve the 3D porous structure of the gel, because the sublimation process without liquid phase can effectively avoid structure collapse and limit structure shrinkage. For freeze-drying technology, the structure of the resulting aerogel could be affected by adjusting the relevant parameters like the temperature and rate of freezing, which could determine the pore size, pore distribution and the number of pores in the resulting aerogel by controlling the size and growth rate of ice crystals.

Buchtova and Budtova conducted a comparative analysis of the impact of various drying techniques on the morphology of cellulose aerogel [41]. Both freeze-drying and vacuum drying at atmospheric pressure resulted in a two dimensional (2D)-sheet-like structure, with vacuum drying producing flat, non-porous aerogels. By contrast, aerogels obtained from supercritical CO_2_ drying exhibited either a cauliflower-like or honeycomb-like structure. The porosity of aerogels produced through supercritical drying and freeze-drying is considerably higher (86–97%) than that of vacuum drying, which typically ranges from 2 to 5%. Similar results were reported by others. Therefore, selecting an appropriate drying technology for aerogel preparation according to the gel type, the desired aerogel morphology, and experimental conditions is necessary.

### 2.3. Carbonization of Biomass-Based CAs

CA can be obtained by carbonization of traditional aerogels. At present, there are two common carbonization methods, that is, high-temperature carbonization and hydrothermal carbonization. High-temperature carbonization means that aerogels are pyrolyzed in an inert gas (nitrogen, argon, etc.) at high temperatures to produce biomass-derived carbon and gas. In the carbonization process, reaction parameters such as reaction temperature, the rate of heating, and holding time need to be strictly controlled. The thermal decomposition of aerogel under inert gas can be divided into four stages: moisture removal stage (25–200 °C), pyrolysis stage (200–500 °C), with the formation of coke and a large amount of gas, amorphous carbon formation stage (500–900 °C) with the breakage of C-C and C-H bonds in coke and the unchaining or aromatization of most of the benzene rings, and the new structure formation stage (above 1000 °C), with the disappearing of C=C bond and reorganization of the internal structure, accompanied by the escaping of a trace of volatile components from the carbon skeleton.

Carbonization temperature significantly impacts the structure and electrical conductivity of CA. Generally, the graphitization degree and the electrical conductivity of biomass carbon materials increase as the temperature increases. However, the graphitization degree decreases sharply when the temperature reaches a certain level [42]. Therefore, biomass-based CA with superior graphitized structure and electrical conductivity can be obtained by regulating the carbonization temperature. In terms of heating rate, higher heating rates tend to produce products with a lower specific surface area [43].

Relatively speaking, hydrothermal carbonization (HTC) is a simple, efficient, economical, and environmentally friendly carbonization method, which generally enables faster formation of carbon from natural biomass under conditions of water as the medium and dissolvent, temperature of 180–250 °C and pressure of 2–10 MPa. The HTC process is mainly divided into three stages: dehydration reaction, demethylation reaction and decarboxylation reaction. Precise control of the pyrolysis products, including structure, composition and morphology, is easily achieved by varying the reaction conditions. Biomass precursors, pyrolysis temperature, holding time, and doping elements are the primary parameters that can influence and modulate HTC.

### 2.4. Functional Modification

To improve the physical or chemical properties of biomass-based aerogels, inorganic materials, especially inorganic nanoparticles with excellent properties, are introduced into biomass materials. 

At present, the metal doping method is the main route for the CA modification by using the carbon aerogel as the skeleton and doping some metal nanostructure to form a composite CA. Metal doping can change the pore structure of CA and make CA chemically active. Ramirez et al., used cellulose acetate-based aerogel as the skeleton structure to prepare Pd-doped aerogel, and the results show that the CA obtained after annealing treatment has a good electrochemical structure and promising applications in the direction of energy storage [44]. Zhang et al., used solvothermal reaction, hydrothermal reaction, and carbonization treatment methods to load MoSe_2_ nanosheets on the surface of cellulose-based CA, and the corresponding MoSe_2_ composite CA showed better hydrogen evolution reaction (HER) electrocatalytic performance than pure MoSe_2_ [45]. 

In addition to metal doping, the heteroatom doping method is another route for CA modification. Incorporating heteroatoms such as N, B, P, S, etc., can effectively adjust the electronic properties, structure, and surface chemical properties of CA. Compared with undoped carbon materials, heteroatom-doped CA materials can effectively enhance the performance of supercapacitors, redox reactions, and lithium-ion batteries, thus showing considerable commercial prospects. In recent years, researchers have successfully prepared a variety of heteroatom-doped CA nanomaterials to meet the growing demand in the energy and catalysis fields. Yang et al., fabricated three-dimensional (3D) interconnected nitrogen-self-doped carbon aerogels (NSCAs) derived from biomass gelatin [46]. The self-contained nitrogen in precursor via pyrolysis process could produce high content of pyridine N and graphitic-N species, which could promote the electrocatalytic activity for ORR. Hu et al., introduced nitrogen-containing functional groups and catalytic active sites by doping the N element in cellulose-based CA, which not only improved the conductivity of electrode materials, but also showed a good CO_2_ adsorption performance [47]. Li et al., reported a highly effective oxygen electrocatalyst based on nitrogen mono-doped carbon nanofiber aerogel, prepared from metal-organic frameworks and bacterial cellulose (BC) complex [48]. This mono-doped carbon material, with N heteroatoms, exhibits exceptional ORR/OER bifunctional activity compared to undoped material. This highlights the efficacy of morphological and structural engineering.

As mentioned previously, the compositions and processes used to construct aerogels can be electrically conductive or catalytic, which makes aerogels viable candidates for the construction of electrocatalysts. As listed in Table 1, the properties of different types of aerogels, such as specific surface area, porosity, and self-supportability, were compared [49]. Good electrocatalysts need to have high activity and durability. Enhancing the intrinsic activity or exposing more reactive sites are both critical for achieving high activity. The former can be achieved by designing special structures within aerogels (e.g., alloys, core-shell structures) or by exploiting synergistic effects (e.g., compounding, doping with heteroatoms) [50]. The latter is fully consistent with aerogel properties, as the high surface area helps the reactants to approach the active surface, and the hierarchical porous structure (micro-, meso-, and macropores) facilitates mass transfer [51]. Improved durability can be achieved by changing the intrinsic properties of the aerogel (i.e., the aerogel backbone) or by exploiting the structural stability of the aerogel. Of all the aerogels that have been reported, CAs seem to be one of the most promising candidates for electrocatalysts.

## 3. Research Progress of Biomass-Based CA for ORR/OER

### 3.1. Mechanism

Currently, the main means for converting and storing clean energy include fuel cells, water electrolysis, ZABs, etc. Oxygen plays a crucial role in the electrode reactions in the electrochemical devices outlined above, either as an active component or as a product. The electrochemical reactions involved in these processes consist of ORR and OER. Specifically, ORR occurs at the cathode of metal–air batteries and fuel cells during discharge, whereas OER happens at the anode of water-splitting devices or during the charge process of metal–air batteries. However, these two reactions need to be carried out at the gas–liquid–solid three-phase boundary, involving multiple electron transfer processes with slow kinetics leading to difficulties of large overpotential, low energy efficiency, and low cycling stability for the air electrode of metal–air batteries. Therefore, to speed up the overall electrochemical kinetics of electrode processes and increase the energy efficiency of associated electrochemical devices for energy storage and conversion, it is crucial to design highly active electrocatalysis for ORR and OER.

#### 3.1.1. ORR Reaction Mechanism

ORR normally occurs at the cathode of electrochemical energy devices. The products in different electrolytes are different, and the electron pathways also differ with the use of different catalysts. Generally, the oxygen reduction reaction involves complex processes such as the adsorption/desorption of oxygen-containing compounds on the catalyst surface, thus the structure of different catalysts will lead to the formation of different oxygen adsorption modes on the catalyst surface and the differences in reaction paths [52]. The two adsorption modes of ORR are bidentate oxygen adsorption and terminal oxygen adsorption. Bidentate oxygen adsorption corresponds to a direct four-electron (4e^−^) pathway without peroxide generation, while terminal oxygen adsorption corresponds to a two-electron (2e^−^) pathway with peroxide generation [53,54,55,56].

(1) Under alkaline electrolytes:

(i) 4e^−^ pathway:

$$O_{2}\left(g\right)+2H_{2}O\left(l\right)+4e^{-}\to 4{OH}^{-}\left(aq\right)\;(E_{0}\;=\;0.4\;V)$$

(ii) 2e^−^ pathway:

$$O_{2}\left(g\right)+H_{2}O\left(l\right)+2e^{-}\to {{HO}_{2}}^{-}\left(aq\right)+{OH}^{-}\left(aq\right)\;(E_{0}\;=\;-0.065\;V)\;{{HO}_{2}}^{-}\left(aq\right)+H_{2}O\left(l\right)+2e^{-}\to {3OH}^{-}\left(aq\right)\;(E_{0}\;=\;0.67\;V)$$

(2) Under acidic electrolytes:

(i) 4e^−^ pathway:

$$O_{2}\left(g\right)+4H^{+}\left(aq\right)+4e^{-}\to 2H_{2}O\left(l\right)\;(E_{0}\;=\;1.229\;V)$$

(ii) 2e^−^ pathway:

$$O_{2}\left(g\right)+2H^{+}\left(aq\right)+2e^{-}\to H_{2}O_{2}\left(l\right)\;(E_{0}\;=\;0.67\;V)\;H_{2}O_{2}\left(l\right)+2H^{+}\left(aq\right)+2e^{-}\to {2H}_{2}O\left(l\right)\;(E_{0}\;=\;1.77\;V)$$

In principle, the 4e^−^ reaction pathway is more favorable for energy conversion. However, the two-electron reaction pathway is easier to generate peroxides and strongly corrode the electrocatalyst, which is not conducive to the stability of metal–air batteries [57]. Therefore, it is expected that the ORR process is all based on the 4e^−^ reaction pathway. However, the 2e^−^ reaction pathway still inevitably occurs due to various factors such as the catalyst activity on the electrode surface, the pH value of the electrolyte, the diffusion rate of oxygen molecules, and the reaction temperature [58].

**Figure 2:**
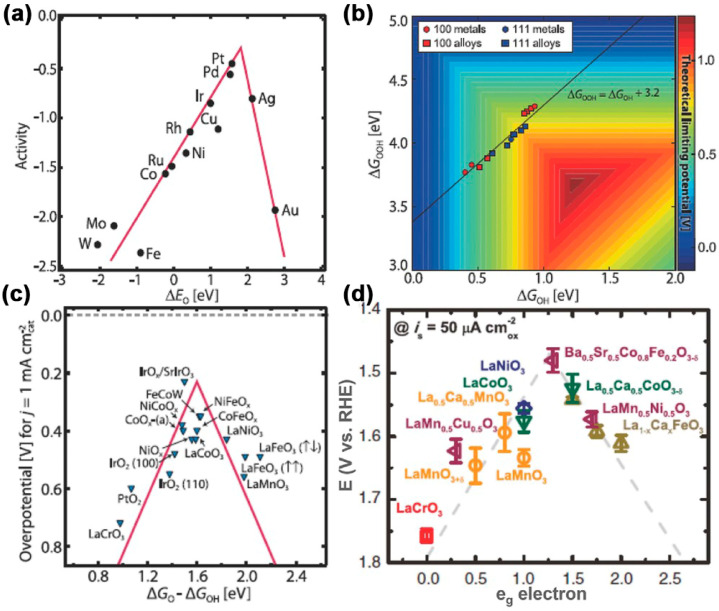
(**a**) ORR volcano plot for metals. (**b**) 2D volcano plot showing the “standard” scaling relationship (ΔGOOH* = ΔGOH* + 3.2) for OOH* and OH* for metals, which also limits the performance of a wide variety of 2D materials. (**c**) OER volcano plot for metal oxides. (**d**) Relation between the OER catalytic activity and the occupancy of the e_g_-symmetry electron of the transition metal (B in ABO_3_). Copyright 2017 by the Wiley from reference [7].

The ORR process involves multi-step electron transfer activities and a complex interconversion of oxygen-containing species intermediates, including OOH*, OH*, and O*. The adsorption process of these intermediates is a crucial part of the ORR kinetics. An ideal electrocatalyst should have a suitable binding energy for the reaction intermediate. Extremely weak intermediate adsorption on the electrode surface will limit the proton and electron transfer of O_2_ molecules. Extremely strong intermediate adsorption on the electrode surface will affect the desorption of H_2_O and even hinder the further adsorption of O_2_ molecules by the active sites on the electrode surface [59,60]. As shown in Figure 2a, researchers have verified that the ORR catalytic activity of various metals has a “volcanic” relationship with the binding energy of O atoms and the noble metal (Pt) is superior to the transition metals (Fe, Co, Ni, etc.) in an ideal position, which means Pt has a higher ORR electrocatalytic activity [7,61,62].

#### 3.1.2. OER Reaction Mechanism

OER is the inverse process of ORR but, in comparison, the OER process is more complex. As mentioned in Section 3.1.1, Pt is almost the ideal pure metal catalyst for ORR. However, the experimental results show that the catalytic effect of Pt on OER is mediocre, demonstrating the difference in reaction mechanism between OER and ORR in the presence of Pt.

OER is a multi-step electron transfer process involving a slow kinetic reaction. With the splitting process of electrocatalytic water molecules, the OER has a high overpotential due to the oxygen generation on the anode, which results in the main energy loss of the OER reaction and thus limits the efficiency of the entire electrochemical system and the industrialization of fuel cells and electrolytic cells.

Metal (even Pt) usually oxidizes because of the high overpotentials required for OER. Therefore, oxygen is precipitated from the surface of high-potential metal oxide rather than from that of the bare metal surface during OER. The OER mechanisms of metal oxides with different surface structures are different [63,64]. In general, the mechanisms of OER on metal oxides in alkaline and acidic electrolytes are as follows:

(1) Under alkaline electrolytes:

$$*+{OH}^{-}(aq)-e^{-}\to {OH}^{*}\;{OH}^{*}+{OH}^{-}(aq)-e^{-}\to O^{*}+H_{2}O(l)\;O^{*}+{OH}^{-}(aq)-e^{-}\to {OOH}^{*}\;{OOH}^{*}+{OH}^{-}(aq)-e^{-}\to O_{2}(g)+2H_{2}O(l)\;\mathrm{Overall}\;\mathrm{reaction}:\;{OH}^{-}(aq)-4e^{-}\to O_{2}(g)+2H_{2}O(l)$$

(2) Under acidic electrolytes:

$$*+H_{2}O(l)-e^{-}\to {OH}^{*}+H^{+}(aq)\;{OH}^{*}+{OH}^{-}-e^{-}\to O^{*}+H_{2}O(l)\;O^{*}+H_{2}O(l)-e^{-}\to {OOH}^{*}+H^{+}(aq)\;{OOH}^{*}+H_{2}O(l)-e^{-}\to *+O_{2}\left(g\right)+H^{+}(aq)$$

In addition, there is another reaction pathway for O_2_ generation in alkaline or acidic electrolytes as shown below:


$${2O}^{*}\to O_{2}\left(g\right)+2\;*$$

Among them, * represents the active site on the catalyst surface, and the binding energy between the catalytic reaction intermediates (O*, OH*, and HOO*) and the catalyst surface significantly impacts the OER activity [65].

Similar to the ORR, the energy state between the intermediates (OOH*, OH*) is utilized as a metric to access the activity of the electrocatalyst [66]. According to the Sabatier principle [67], too weak bonding strength between the electrocatalyst surface and O is not conducive to forming the intermediate product (OH*), and a large bonding strength between the electrocatalyst surface and O will prevent OH* from reacting further to produce OOH*. Therefore, increasing the OER site’s catalytic activity benefits only from moderate bonding strength. To date, metal oxides have been the most popular electrocatalysts for OER. Figure 2c shows a “volcano-shaped” curve of the binding energy relationship between metal oxide catalysts and intermediates, including rutile, perovskite, spinel, rock salt, bicarbonate, and double oxide. Since the enthalpy change in all four steps of OER increases, whether the step with the largest enthalpy increase can be carried out or not determines whether the whole reaction can be completed. The standard potential of OER is 1.23 V, but in the actual process, due to the impurities and other factors, there is inevitably an overpotential, so the actual potential of OER is much higher than 1.23 V.

### 3.2. Non-Metal-Doped Biomass-Based CA Modified Catalyst

The non-metal-doped biomass-based CA catalyst has no metal nanoparticles, and the carbon aerogel itself is a catalyst without impregnation reduction and other process steps. In general, the preparation method is simple and low cost, thus showing great electrocatalytic potential, especially in ORR. In addition to the characteristics mentioned above, the other advantages are listed as follows:

(1) Due to the characteristics of biomass itself, the CA generated during pyrolysis can directly introduce N into the aerogels without the addition of any additives to obtain N-doped carbon aerogels, which would enhance the electron transfer and improve the ORR catalytic activity [68,69].
(2) Also, the multiscale porosity of CA is a crucial factor in determining their ORR catalytic activity. Micropores can provide a larger surface area and more active sites, which are beneficial for catalytic reactions. Mesopores can offer better transportation channels, facilitating the diffusion and transfer of reactants and products. Macropores can provide better fluidity, enabling the uniform distribution of reactants and regeneration of the catalyst [70,71].
(3) The large specific surface area and 3D hierarchical porous structure are conducive to the full exposure of the ORR active sites.
(4) The surface structure and inherent hydrophobicity of CA ensures the high stability of the enlarged liquid/gas/solid three-phase interface, making it favorable to the increase in the number of active sites and the improvement in the ORR reaction efficiency [72,73].

In order to promote the activity and stability of catalysts, heteroatom doping, including N, P, S, B, etc., is usually performed in biomass-based CA. The presence of heteroatoms doped in the carbon skeleton alters the charge density distribution and bond length of carbon atoms in the vicinity of the dopant, which leads to the defects and structural distortions that improve the electrochemical activity. The heteroatom doping combined with the 3D porous structure supplies a great amount of catalytic active sites along with the formation of a stable ion diffusion network.

#### 3.2.1. Mono-Doped CA

**Figure 3:**
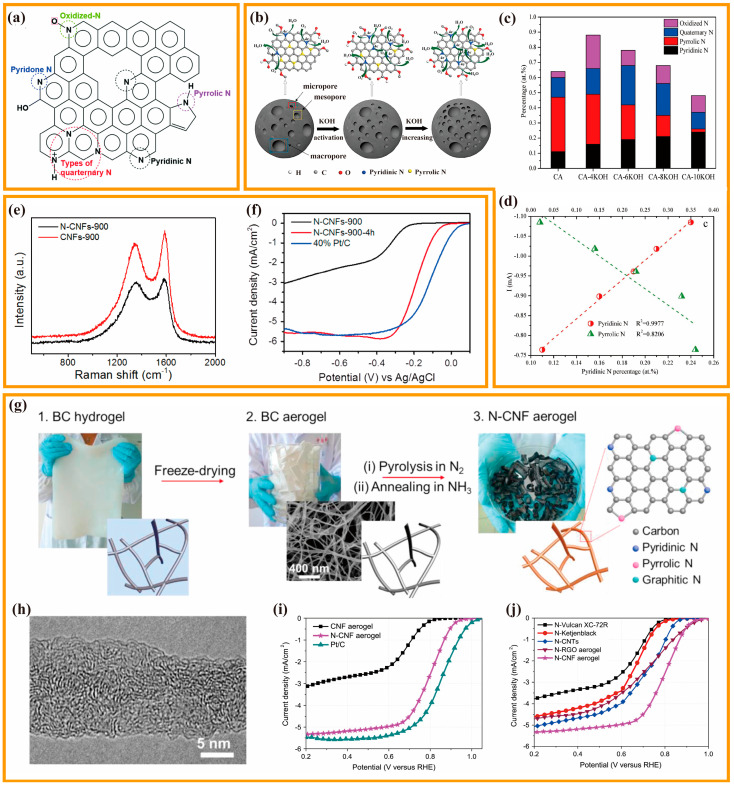
(**a**) Several different types of N-configurations in N-doped carbon. Copyright 2021 by the Royal Society of Chemistry from reference [77]. (**b**) Schematic mechanism of the possible role of KOH in ORR. (**c**) Changes in different N-configuration contents as KOH increases. (**d**) Linear relationship between the content and ORR activity of pyridine-N and pyrrole-N, respectively. Copyright 2018 by the Elsevier from reference [78]. (**e**) Raman spectroscopy before and after nitrogen doping. (**f**) Effect of activation time on the ORR activity of N-CNF by LSV curves. Copyright 2016 by the Elsevier from reference [79]. (**g**) Schematic diagram of the synthesis of N-CNF aerogels. (**h**) HRTEM image of N-CNF. (**i**) ORR curves of N-CNF aerogel and comparison catalysts. (**j**) ORR curves of N-CNF aerogel and other NH_3_-treated carbon materials. Copyright 2015 by the Elsevier from reference [80].

Among all the heteroatom-doped carbon options, N-doped nanocarbon has garnered the most attention since N atoms possess a comparable atomic radius to C atoms but have one additional electron compared to C atoms [74]. After putting the heteroatom into the carbon skeleton, the former can prevent lattice mismatch, whereas the latter favors electron-involved reactions such as ORR [75,76]. In most circumstances, these two benefits provide N-doped carbons with higher ORR activity and stability than other non-metal heteroatom-doped carbons. Generally, several different N configurations could be formed by anchoring N atoms into sp^2^ hybridized carbon materials, i.e., pyrrole-N, pyridine-N, graphitic-N (quaternary-N), and oxidized-N (Figure 3a) [77]. Graphitic-N refers to the combination of an N atom with the three adjacent C atoms in a hexagonal lattice, usually situated at the center of the carbon material. By contrast, pyrrole-N and pyridine-N are located at the periphery of the carbon material, with pyridine-N forming a secondary coordination and pyrrole-N consisting of a pentagonal structure bonding with two adjacent carbon atoms. However, it is still controversial which configuration in N-doped carbon is the true active site.

To gain a more thorough recognition of the catalytic activity mechanism of N-doping, Tian et al., used glucose, aniline, and (NH_4_)_2_S_2_O_8_ as carbon and nitrogen sources to prepare N-doped CA by one-pot template-free method and hydrothermal method. N-doped CA was chemically activated with KOH as an ORR catalyst for microbial fuel cells [78]. Figure 3b shows a schematic mechanism of the possible role of KOH in ORR. The results show that KOH activation regulated the contents of N-containing and O-containing functional groups. With the increase in KOH addition, the content of pyridine-N increased but that of pyrrolic-N decreased (Figure 3c). In addition, KOH activation also led to the increase in the contents of C-O-C and COOH. The electrochemical test demonstrated that pyridine-N had a positive linear relationship with electrochemical activity, while pyrrolic-N showed an inverse trend. The increase in pyrrolic-N will result in the reduction in the electrochemical activity (Figure 3d), and a small amount of C-O-C and COOH is conducive to the reduction in the H_2_O_2_ yield, thereby improving the efficiency of fuel cells. Song et al., introduced a template-directed hydrothermal carbonization method for the preparation of N-doped carbonaceous nanofibrous material aerogels (N-CNFs) [79]. Specifically, ultrafine tellurium nanofibers were used as templates, and glucosamine served as both C and N sources to prepare N-CNFs. Then, high temperature treatment and CO_2_ activation were conducted to prepare N-CNFs CA. It was found that introducing the N element leads to more defects in the carbon structure and reduced graphitization (Figure 3e), thus increasing the catalyst ORR activity. This research group also investigated the effects of activation time on N-CNFs. The highest ORR activity could be observed at the activation time of 4 h for CO_2_ (Figure 3f), which shows great relationships with the equilibrium among the porosity, N element content and conductivity under this condition. Liang et al., then obtained N-doped carbon aerogels (N-CNF) by freeze-drying, pyrolysis, and activation by annealing under NH_3_ using green BC as the raw material (Figure 3g) [80]. High resolution transmission electron microscopy (HRTEM) images show that the N-CNF is made up of amorphous and flawed carbon structures together with layers of graphene that are irregularly orientated (Figure 3h). At high temperatures, free radicals generated by NH_3_ decomposition can etch away carbon fragments, leaving newly created micropores that contain nitrogen-containing active sites. Additional nitrogen-containing groups are also produced owing to the substitution reaction between oxygen-containing species on the carbon surface and NH_3_. The resulting doped-nitrogen flaws in the edges and planes of graphene can function as ORR active sites. Therefore, N-CNF shows remarkable ORR performance, displaying an onset potential (E_onset_) of 0.83 V and a half-wave potential (E_1/2_) of 0.80 V, which is merely 50 mV less active than that of the Pt/C catalyst (Figure 3i). N-CNF CAs had a higher ORR activity than that of NH_3_-treated carbon black, CNT, and reduced graphene oxide aerogels, as well as the majority of reported metal-free catalysts (Figure 3j). Owing to the distinctive 3D nanofiber network structure of CNF CA, it is easier to have intimate contact with gaseous NH_3_ molecules than other carbon materials, leading to a more efficient activation process and a more uniform distribution of functionalized sites. Also, ZAB made of N-CNF has an open-circuit voltage of 1.50 V, a specific capacity of 615 mAh·g^−1^, and a gravimetric energy density of 760 Wh·kg^−1^, which is comparable to that made of Pt/C. The ORR and OER performance of all the catalysts mentioned above and also below are summarized in Table 2.

Other heteroatoms, including B, P, and S, are also involved in the modulation of sp2 carbon besides nitrogen doping, considerably extending the dimensionality of the regulation. However, we found little about single element doping of non-metal CA catalysts other than N-doping. Nevertheless, some experimental work on co-doping can be found, which will be presented in the next section.

#### 3.2.2. Dual-Doped CAs

The significant impact of single heteroatom-doped metal-free catalysts on the ORR has motivated researchers to develop a range of catalysts that incorporate two or more heteroatoms simultaneously into carbon, which aims to harness the synergistic effect of different elements to further improve electrocatalytic activity.

**Figure 4:**
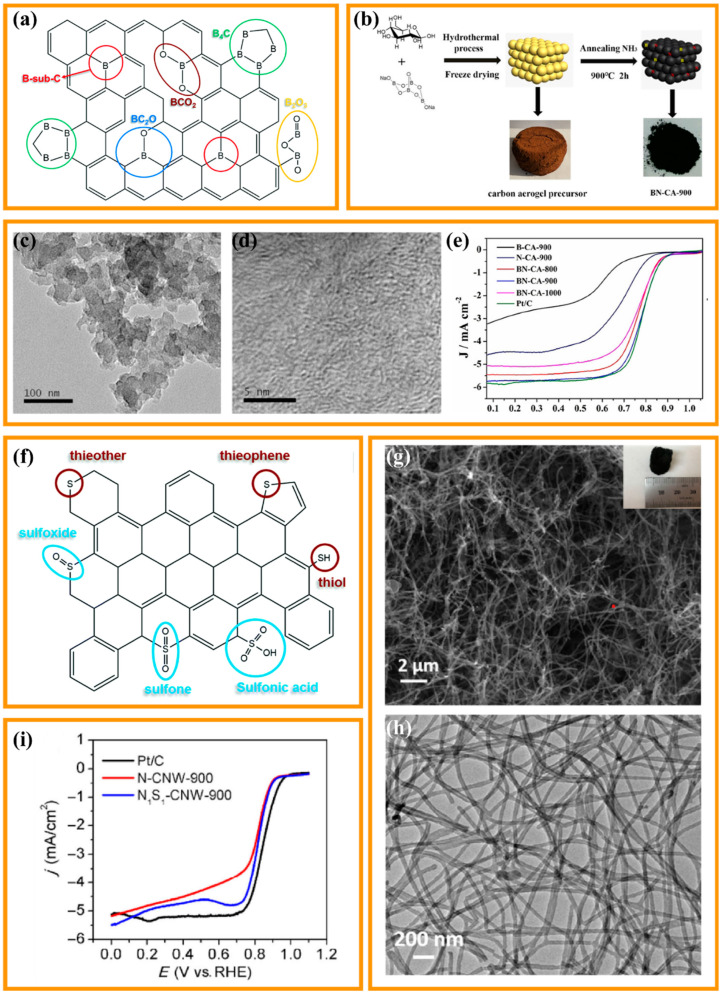
(**a**) Several different types of B-configurations in B-doped carbon. Copyright 2021 by the Royal Society of Chemistry from reference [77]. (**b**) Schematic diagram of the synthesis of BN−CA−900. (**c**) TEM and (**d**) HRTEM image of BN−CA−900. (**e**) ORR curves of BN−CA−900. Copyright 2019 by the Wiley from reference [80]. (**f**) Several different types of S-configurations in S-doped carbon. Copyright 2021 by the Royal Society of Chemistry from reference [77]. (**g**) SEM and (**h**) TEM image of N_1_S_1_−CNW−900. (**i**) ORR curves of N_1_S_1_−CNW−900. Copyright 2016 by the Springer Link from reference [82].

In contrast to the carbon materials doped with N heteroatom, B atom doping will form positively charged sites (B*) for oxygen adsorption due to the low electronegativity of B atoms (2.04). Additionally, B-doping will disrupt the electroneutrality and spin distribution of the adjacent C atom, which is favorable for the electron transfer to the C atom and thus promotes the creation of B* sites and C=B covalent bonds (Figure 4a) [77]. Distinct from the N atom with an excess of electrons, the B dopant is electron-deficient and can change the electroneutral state of the sp^2^ hybridized carbon, thus creating a positively charged position favorable for oxygen adsorption. Despite having strong electrocatalytic activity, B-doped carbon has received relatively little study attention up until this point. The main reason might be a scarcity of effective boron sources and the difficulty in boron preparation. Better catalytic activity is demonstrated by N and B co-doped carbon materials when compared to single B-doping materials, primarily because B and N have a synergistic impact when placed on the sp2 carbon substrate, respectively. Yu et al., used cheap, common, and non-polluting glucose and borax as raw materials to synthesize B, N co-doped non-metallic carbon aerogel catalyst (BN-CA-900) by hydrothermal treatment and carbonization process under NH_3_ atmosphere (Figure 4b) [81]. Here, pyrolysis under the NH_3_ atmosphere introduces heteroatomic N into the carbon material and leads to the creation of carbon defects (Figure 4c,d). Additionally, due to the contrasting properties of N-doping and B-doping, B and N co-doping can synergistically regulate the charge density of carbon materials and exhibit a synergistic effect on the ORR catalytic process (Figure 4e), thus leading to the formation of more defects to increase the number of active sites. Specifically, N has a greater electronegativity than C. Therefore, N doping generates positive charges on adjacent carbon, thus having a promotion effect on oxygen reduction reactions. On the contrary, B is less electronegative than C. The conjugated carbon π system has free 2pz orbitals to extract electrons, thus facilitating O_2_ adsorption and reduction in positively charged B sites. 

For nonmetallic S atoms, S doping enhances catalysis by tuning the π-electrons of the sp^2^ carbon instead of destroying the electroneutrality, as its electronegativity (2.58) is close to that of C (2.55), but its outermost orbitals do not match with C [77]. Thus, S doping can modulate the redistribution of the spin density of surrounding C atoms but not the redistribution of charge. It was found that –C–S–C– and –C=S– groups could be formed by the interaction between S atoms and C atoms (Figure 4f), with the presence of S atoms in the thiophene phase, which was verified as the active center of OER in electrocatalysis C atoms with high spin density. Single S-doping has been rarely reported, while S and N co-doped carbon-containing materials have been more extensively explored due to the synergistic effect of double doping, which favors the adsorption of oxygen and its associated species, especially when S and N atoms are bonded with the same C atom. Fu et al., proposed a cost-effective approach for the preparation of N and S co-doped carbon nanowire aerogels using the hard template method [82]. A combination of hydrothermal treatment and carbonization was employed to synthesize N_1_S_1_-CNW-900. The extensive specific surface area and porous structure (Figure 4g,h) ensured an adequate exposure of the active site as well as good mass transfer and gas diffusion by ORR. Furthermore, X-ray photoelectron spectroscopy (XPS) analysis demonstrated that N and S elements may be effectively doped regardless of the heat treatment in N_1_S_1_-CNW-900. It is shown that co-doping carbon materials with N and S to create co-doped catalysts generates a multitude of active carbon atoms, due to the asymmetric spin and charge density caused by N and S doping. In addition, the doping levels of N and S, and the N/S ratio, play a crucial role in regulating the electronic structure of the resulting catalysts, thereby accurately regulating their catalytic performance. Due to its distinctive chemical and structural properties, the combined N_1_S_1_-CNW-900 aerogel shows comparable electrocatalytic activity for ORR (E_onset_ = 0.905 V, E_1/2_ = 0.838 V), comparable to commercial Pt/C (E_onset_ = 0.931 V, E_1/2_ = 0.851 V) (Figure 4i). 

### 3.3. Biomass-Based CA Supported Non-Noble (Transition) Metal Modified Catalysts

Currently, Pt and Pt alloys are used as fuel cell catalysts with the best catalytic performance. However, the precious metal Pt has high cost and limited reserves limitations, which greatly hinder the large-scale commercial application of fuel cells. Therefore, it is crucial and meaningful in designing and preparing cost-effective and high-performance non-noble metal ORR/OER catalysts. Transition metals show excellent catalytic activity, but poor oxidation resistance. It is found that the composite of transition metal particles and carbon materials exhibits excellent catalytic performance as a bifunctional catalyst for ORR and OER [83]. Within this composite structure, transition metals can lead to an elevation in the graphitization degree of carbon materials during carbonization. Furthermore, the carbon materials coating on the surface can effectively hinder metal agglomeration to ensure dispersion and promote electron transfer [84].

#### 3.3.1. Single Atom

In 2011, the research team of Prof. Tao Zhang successfully synthesized a single-atom Pt catalyst (Pt_1_/FeO_x_) [85]. Various instrumental test characterization methods verified the presence of single-atom Pt. Pt_1_/FeO_x_ showed excellent activity, stability, and selectivity in the CO oxidation reaction and CO selective oxidation reaction under hydrogen-rich atmosphere. This work introduced the concept of “single-atom catalysis” for the first time, and set off a research upsurge of single-atom catalysis worldwide. Single-atom catalysts are a type of supported catalysts that only use isolated single atoms as catalytic active centers, showing nearly 100% atom utilization and outstanding catalytic activity. It is well known that maximizing the amount of exposed catalyst active sites or increasing the density of the catalyst active site can optimize the catalytic performance. The active centers in single-atom catalysts are the metals that are uniformly dispersed on the support in the configuration of individual atoms, which is conducive to the high activity and selectivity of catalytic reactions, and also provides a new solution for revealing the catalytic mechanism [80,86].

M-N-C catalysts (M represents transition metal) are non-noble metal single-atom catalysts with the highest redox activity at present. Such catalysts have advantages such as cost-effectiveness, super activity, high stability, and excellent resistance to methanol poisoning. M-N-C catalysts are usually formed through the thermal decomposition of metal, nitrogen, and carbon sources at high temperatures. There has been no consensus on the active sites of M-N-C catalysts till now. One viewpoint is that nitrogen-doped carbon is the active site, and the metal ion center has a catalytic effect on the formation of various N-containing functional groups. Another one viewpoint believes that M-N_X_ is the active site. From these two viewpoints, the ORR performance of M-N-C shows great relationships with carbon support, metal source, nitrogen source, and heat treatment temperature. Numerous studies have demonstrated that the ORR performance of Fe and Co exceeds that of other transition metals (Zn, Ni, Mn, Cu, Cr), and carbon materials modified with Fe-N_X_ show higher activity than commercial Pt/C.

**Figure 5:**
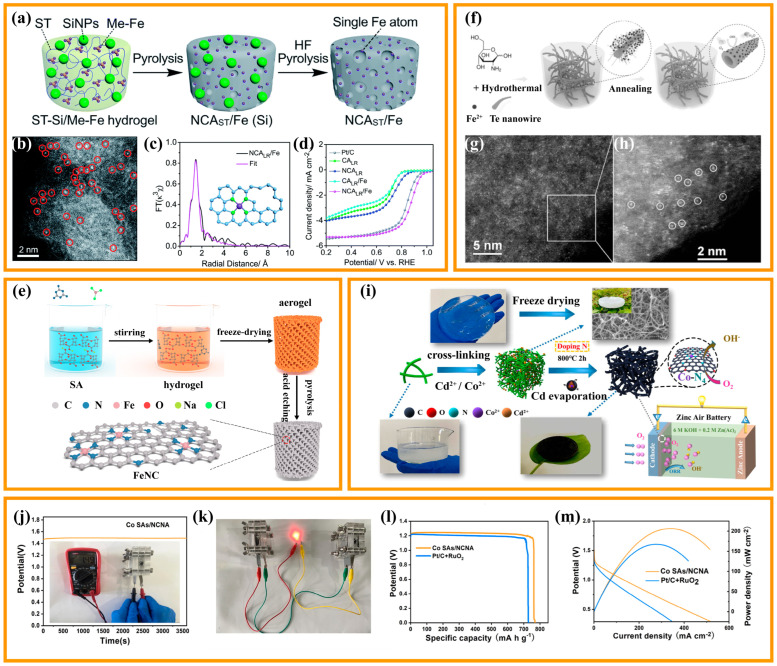
(**a**) Schematic diagram for the synthesis of NCA_ST_/Fe. (**b**) HAADF−STEM image of the NCA_LR_/Fe aerogel where the red circles are marked with Fe single atoms. (**c**) Corresponding EXAFS spectrum and fitting curve for NCA_LR_/Fe. (**d**) ORR curves of NCA_LR_/Fe and comparison samples. Copyright 2019 by the Royal Society of Chemistry from reference [88]. (**e**) Schematic diagram for the synthesis procedure of the FeNC catalysts. Copyright 2021 by the Elsevier from reference [89]. (**f**) Schematic diagram for the synthesis procedure of Fe–N−CNTAs. (**g**) TEM images of Fe-N-CNTA and (**h**) the white circles are marked with Fe single atoms. Copyright 2017 by the Wiley from reference [90]. (**i**) Schematic diagram of the synthesis of Co-SAs/NCNA. (**j**) Open-circuit voltage of the Co SAs/NCNA-based ZAB. (**k**) Photo of the lighted LED by two ZABs in series. (**l**) Galvanostatic discharge curves and (**m**) polarization/power density curves of Co SAs/NCNA-based ZAB and Pt/C + RuO_2_-based ZAB. Copyright 2023 by the Elsevier from reference [91].

The researchers found that a multitude of micropores and mesopores can be obtained by ammonia treatment, water vapor treatment, etching, and templating agent pore creation to enhance the catalyst transfer rate for mass and electron transfer. Meanwhile, these modulations significantly expand the specific surface area of the catalyst and thus supply more active sites for catalytic reactions. Single-atom structures at the edges of defects typically have higher catalytic activity than in-plane structures. With a deep understanding of defect modulation, it was found that defect modulation could improve the catalytic activity of catalysts, because it can modulate the electronic structure of catalytic-type centers, and thus modulate the adsorption energy of catalysts for ORR reaction intermediates (O_2_*, OH*, OOH*). Wang et al., modified CA by simple alkali etching (KOH) and high-temperature treatment, to prepare oxygen reduction catalysts with 3D porous Fe, N-doped CAs (Fe-N-CA) using melamine as the nitrogen source and ferrocene as the iron source [87]. KOH high-temperature etching can successfully create micropores and mesopores, which is favorable for the mass transfer and the exposure of active sites, and thus enhances the catalysis. It was shown that the activity and stability of Fe-N-CA-800 are close to those of commercial Pt/C catalysts under alkaline conditions (E_onset_ = 0.918 V, E_1/2_ = 0.798 V), and the reaction was ideal for a four-electron pathway. SCN-poisoning experiments yielded Fe mainly in the form of Fe-N_X_ active sites, and the doping of trace Fe (0.6 wt.%) led to a dramatic increase in the ORR activity. In previous studies on Fe-N-C single-atom catalysts, the single atom of the Fe-N_4_ structure is usually regarded as the catalytically active center of ORR and has become a research hotspot in this field. He et al., prepared 3D CA-loaded Fe single-atom catalysts (NCA_LR_/Fe) using biomass hydrogels as precursors (Figure 5a) [88]. BET, XPS, XAS, and Mössbauer characterization showed that microporous defects were formed in situ in the porous CA (Figure 5b), resulting in the capture and stabilization of individual Fe atoms and the formation of highly active FeN_4_ catalytic centers (Figure 5c). Microporous defects are formed during the high temperature graphitization of starch promoted by melamine. The resulting microporous defects help to trap and stabilize the mobile Fe atoms by physicochemical interactions. In addition, Fe atoms exist mainly near the micropore edges and are easily accessible during the catalytic reaction due to the plugging effect of the single metal site formed in situ. Electrochemical tests showed that NCA_LR_/Fe monoatomic carbon aerogel has an onset potential of up to 1.05 V and a half-wave potential of up to 0.88 V in alkaline media (Figure 5d), and shows excellent methanol tolerance, durability, and redox activity. Remarkably, when the CAs are used as the cathode catalyst in metal–air batteries, the battery achieves an ultrahigh open-circuit voltage of 1.81 V, large power density of 181.1 mW·cm^−2^, and stable discharge voltage of 1.70 V, markedly better than those with commercial Pt/C as the cathode catalyst. Hao et al., proposed a feasible and facile strategy for biomass aerogels pyrolysis, to prepare single-atom Fe catalysts (FeNC) by anchoring iron ions through ligand reactions (Figure 5e) [89]. A high-angle annular dark-field scanning transmission election microscope (HAADFS-TEM) clearly identifies the presence of single-atom Fe and shows that sodium alginate (SA) is an excellent precursor for the uniform dispersion of Fe atoms on the surface of carbon materials. Combined with the results of the extended X-ray absorption fine structure (EXAFS) test, it can be demonstrated that Fe species dispersed as FeN_4_ exhibit the best ORR performance (E_1/2_ = 0.88 V), even better than commercial Pt/C. The outstanding ORR activity of FeNC-900-8 is attributed to the copious micropores and expansive specific surface area (1364 m^2^/g), which allows the full exposure of active sites to ensure the effective participation in the catalytic reaction. As a cathode catalyst for ZABs, FeNC-900-8 also exhibits exceptional performance. The FeNC-900-8-based ZAB reaches an open circuit voltage of 1.67 V and provides a peak power density of 124.9 mW·cm^−1^. At 5 mA·cm^−1^, the specific capacity was calculated to be 816.4 mA h/g, which is close to the theoretical capacity. These characteristics hold significant promise for deployment in energy conversion devices.

Besides the intrinsic activity modulation of the catalyst for full exposure of the reactive sites, the supply of more accessible sites for reaction also plays a crucial role. In general, catalytic reactions occur only if the reactants can reach the catalytic reactive sites. Hence, the exposure of the reactive sites to the catalyst surface as much as possible increases the density of reactive sites and enhances the contact between the catalyst and the reactants which, in turn, enhances the reaction rate. The design of the exposed active site mainly relies on the design of the catalyst geometry, dimensionality, and pore structure. In order to expose more active sites, templating agents are usually used in the material synthesis to gain a shell structure or hollow structure of the exposed sites. Zhu et al., prepared a carbon nanotube aerogel (Fe-N-CNTA) loaded with Fe-monoatoms using tellurium nanowires as a template, and nitrogen-containing small molecules (glucosamine hydrochloride) and inorganic salts (NH_4_Fe(SO_4_)_2_·6H_2_O) as a nitrogen source in a one-step hydrothermal treatment combined with heat treatment (Figure 5f) [90]. The catalyst with only 0.09 at% Fe content exhibited better ORR activity (E_onset_ = 0.97 V, E_1/2_ = 0.88 V) than Pt/C (E_onset_ = 0.96 V, E_1/2_ = 0.86 V), which was mainly due to the unsaturated coordination state of the active center and the stable interaction with the carrier. In conclusion, the porous structure, characterized by a unique network of hollow nanowires, provides a large surface area as well as the ability to fully expose the active sites, which facilitates mass and electron transfer. The uniform single-atom distribution of Fe ions in the carbon matrix (Figure 5g,h) ensures the homogeneity of the active components. It is conducive to boosting the catalytic efficiency of the catalyst. The above-mentioned factors favor Fe-N-CNTAs as promising ORR electrocatalysts for fuel cells.

Although Fe single-atom catalysts exhibit better catalytic activity and fuel cell performance among non-precious metal catalysts, a two-electron side reaction is inevitably carried out in the actual process to produce hydrogen peroxide. The interaction between hydrogen peroxide and Fe^2+^ or Fe^3+^ leads to the production of hydroxyl radicals by the Fenton reaction. These active hydroxyl radicals will attack the surrounding carbon or metal active centers, and in turn cause performance degradation. Meanwhile, these active hydroxyl radicals will also attack the polymer diaphragm, leading to battery performance degradation. Therefore, non-precious metal single-atom catalysts with weak Fenton effect and high activity have been expected. Shen et al., proposed a feasible method to construct metal-chelated TOCNFs-Cd^2+^/Co^2+^ aerogel precursors using cellulose nanofibers, and synthesized N-doped carbon nanofiber aerogel-based single cobalt atom catalysts (Co-SAs/NCNA) by eliminating volatile metal cadmium and doping additional nitrogen during direct calcination (Figure 5i) [91]. Due to the presence of a multitude of exposed active metal atomic sites and a conductive network with high-speed electron/mass transfer channels within the 3D porous CA, the Co-SAs/NCNA produced shows higher ORR kinetics (E_onset_ = 0.98 V, E_1/2_ = 0.86 V) under alkaline conditions and has excellent performance (206 mW·cm^−2^ of high maximum power density, 769 mAh·g^−1^ of specific capacity) when used as air cathodes in ZAB devices (Figure 5j–m).

In conclusion, the M-N-C class of single-atom electrocatalysts represents a hopeful possibility for advancing the next generation of bifunctional oxygen electrocatalysts, which can realize the effective loading of atomic-sized active sites into carbon materials. In terms of intrinsic activity, the exposed active sites for M-N-C electrocatalysts are far more than those for most carbon-based materials (e.g., non-metal doping). From the perspective of atomic utilization, the dispersion of active sites in atomic form is one of the most advanced techniques to reach the theoretical limit. However, the catalysts of M-N-C type still face some serious issues, such as insufficient effective metal loading, limited stability, and an inadequate comprehension of the mechanism and active sites. Aimed at solving the shortage of Pt resources, reducing the cost of ZAB batteries, and promoting the commercial application of ZAB batteries, it is necessary and meaningful to develop functional composite M-N-C catalysts, explore the relationship between their electrochemical performance and structure, improve their corrosion resistance and stability, and to search for cost-effective and high-performance M-N-C catalysts that can replace Pt-based catalysts. 

#### 3.3.2. Transition Metal Compounds

Transition metal compound catalysts mainly include transition group metal oxides, sulfides, phosphides, nitrides, etc. The unique electronic structure generated by the interaction between the d orbitals of the transition metals and the p orbitals of the primary group elements facilitate electrocatalytic reactions. More importantly, transition metal compound catalysts show obvious advantages such as wide availability, versatility, low cost, being environmentally friendly, and involving simple preparation, and thus have attracted widespread attention. Therefore, the modulation of chemical composition and morphological structure of various transition metal compound electrocatalysts has been carried out to investigate how to further improve the electrocatalytic performance of transition metal compound electrocatalysts.

**Figure 6:**
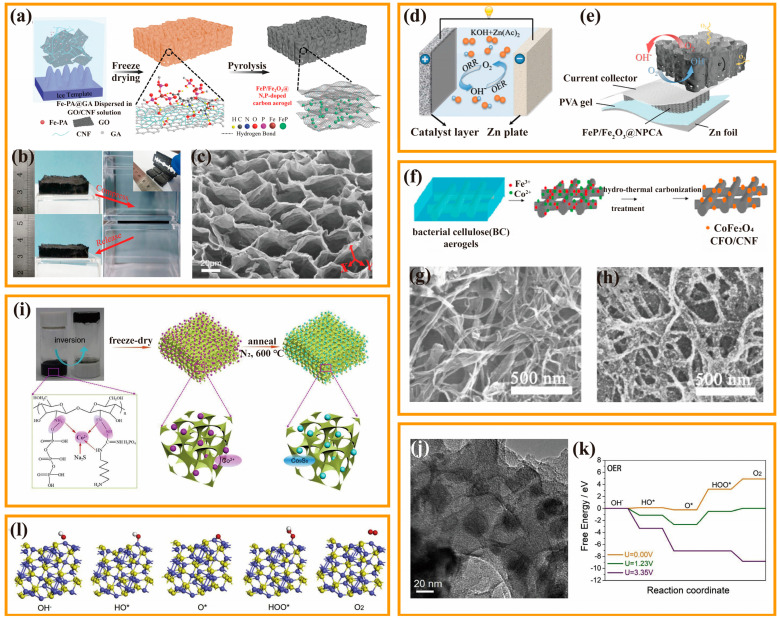
(**a**) Schematic diagram for synthesis of the FeP/Fe_2_O_3_@NPCA. (**b**) Comparison photos of FeP/Fe_2_O_3_@NPCA before and after compression. (**c**) SEM image of FeP/Fe_2_O_3_@NPCA. (**d**,**e**) Schematic diagram of aqueous rechargeable and solid-state ZAB. Copyright 2020 by the Wiley from reference [92]. (**f**) Schematic diagram for the synthesis of CFO/CNF nanocomposite. (**g**,**h**) SEM images of CFO/CNF nanocomposite. Copyright 2016 by the Elsevier from reference [93]. (**i**) Schematic diagram for the synthesis of Co_9_S_8_/N, P−APC. (**j**) TEM image of Co_9_S_8_/N, P−APC. (**k**) OER free energy diagrams at different potentials. (**l**) Several different reaction intermediates configurations. Copyright 2019 by the Elsevier from reference [16].

Metal oxides hold great promise as electrocatalysts due to their easy accessibility, ability to tune their morphological structure, long-term stability, and high theoretical catalytic activity. However, the further improvement in their catalytic activity is limited because of the weak electrical conductivity of metal oxides and their tendency to deactivate at high currents. Combining metal oxides and highly conductive carbon compounds is considered a particularly successful tactic to overcome the above challenges. The strong interaction between carbon material and transition metal oxide can firmly anchor the metal oxide. Therefore, the overall conductivity of the electrocatalyst is improved, the dispersion of the metal oxide is enhanced, and the mass transfer of the catalyst during the reaction is enhanced. Additionally, the carbon substrate can shield the transition metal oxide from electrolyte erosion during the catalytic process, and the electronic structure is altered because of the cladding structure, which has a synergistic effect of improving the catalytic activity. Peng et al., designed a 3D self-supporting flexible air electrode material using nanocellulose as raw material [92]. Different from the traditional complex bottom-up synthesis method, a facile strategy for Fe-based N,P-doped CA is proposed (Figure 6a). A 3D honeycomb-like bulk CA electrode material with the in situ growth of FeP/Fe_2_O_3_ nanoparticles was synthesized by directional freeze-drying and carbonization treatment. The resulting CA had good mechanical stability and three-dimensional porous structure (Figure 6b,c), enabling the efficient diffusion of gases and electrolytes. Benefiting from the synergistic effect between non-noble metal compounds and heteroatom-doped carbon substrates, FeP/Fe_2_O_3_@NPCA exhibits remarkable ORR and OER performances (ΔV = 0.79 V), small overpotential, and high stability. Based on the above advantages, iron-based modified wood-derived CAs show great potential to be used as a promising air cathode material in conventional aqueous ZAB, or flow-type ZAB and flexible solid-state ZAB (Figure 6d,e). The research conducted in this study is a significant breakthrough in the areas of biomass cellulose-derived materials and electrocatalysis. The development of free-standing CA-based electrodes holds vast potential for diverse energy conversion and storage applications. In addition to single metal oxides, composite metal oxides are also used as transition metal oxide catalysts. Typically, composite metal oxides have a spinel structure with the chemical formula AB_2_O_4_. These metal oxides meet the requirements of ORR and OER and could facilitate electron transfer due to the synergistic effect between divalent and trivalent ions. They can further upgrade the electrochemical properties by complexation with carbon carriers and offer a wide range of different structural forms depending on the rational choice of preparative body conditions and prerequisites. Liu et al., used BC as a carbon fiber precursor to prepare a 3D network CoFe_2_O_4_/CNF composite catalyst with ORR and OER bifunctional catalytic activity using the hydrothermal method (Figure 6f) [93]. After incineration and carbonization under a N_2_ atmosphere, the freeze-dried BC aerogel shows the structural properties such as a large specific surface area (545.03 m^2^·g^−1^) and a layered porous structure. CoFe_2_O_4_/CNF composites synthesized using the hydrothermal method have a high catalytic performance and catalytic stability. On the one hand, CNF has the ability to anchor the functional groups of CoFe_2_O_4_ nanoparticles without any pretreatment, along with the uniform dispersion of CoFe_2_O_4_ nanoparticles on the CNF surface (Figure 6g). On the other hand, the 3D network and mesoporous structure of CoFe_2_O_4_/CNF composites provide more catalytic sites for electrocatalytic reactions and improve the transfer of reactants, electrolytes, and electrons (Figure 6h).

Metal nitrides represent another type of electrocatalysts that exhibit remarkable ORR catalytic performance. Using carbon materials as substrates can provide highly conductive support for the damascene of metal nitrides and serve as carbon sources for preparing nitrogen-doped carbon matrices, or enhance material activity through the synergistic effect between metal nitrides and carbon. Li et al., designed a new ORR catalyst with cocoon as the precursor for the synthesis of ternary doped porous CA with heteroatoms (N, S, and Fe) [94]. The aerogels have a variety of active sites, including Fe_3_C, FeN_x_, and various complexes. Notably, the obtained ternary doped porous CA catalyst HDCA-800, has a large specific surface area (714.4 m^2^·g^−1^), excellent electrocatalytic activity (E_onset_ = 0.94 V; E_1/2_ = 0.79 V), and a high confinement current density (3.80 mA·cm^−2^) via a nearly 4e^−^ reduction pathway, comparable to the base reduction pathways of commercial Pt/C catalysts in neutral media. 

Metal sulfide catalysts are mainly transition metal sulfides, such as FeS, CoS_2_, NiS_2_, etc. Previous studies indicated that increasing the interface, vacancies, and lattice defects of metal sulfides can help increase the catalytically active sites and improve the catalytic performance of metal sulfides. Hu et al., proposed an efficient method to enable the homogeneous anchoring of Co_9_S_8_ particles in CA [16]. Specifically, polyhexamethy-leneguanidine phosphate and sodium tripolyphosphate were used as precursors to prepare Co_9_S_8_-doped CA by sol-gel polymerization (Figure 6i). Compared with other supported catalysts derived from the impregnation method, the newly developed material shows an obvious advantage, that is, the Co_9_S_8_ particles are uniformly anchored in the porous N,P co-doped carbon substrate (Co_9_S_8_/N,P-APC) (Figure 6j). When used as a catalyst, Co_9_S_8_/N,P-APC exhibited E_onset_ = 0.89 V and E_1/2_ = 0.78 V during ORR, and an overpotential of 363 mV at 10 mA·cm^−2^ during OER. The calculation of free energy based on density functional theory proves that the OER activity correlates with the energy of OER intermediates (e.g., OH*, O*, and OOH*) (Figure 6k,l). In the above two processes, the unique honeycomb-like porous structure facilitates the intimate contact of the electrolyte and the exposed Co_9_S_8_. Moreover, the electrons’ transfer along the 3D porous channel is quicker, and is thus conducive to effectively utilizing the active Co_9_S_8_ particles.

#### 3.3.3. Metal Alloy

With the deepening of research, researchers have prepared a series of catalysts by doping bimetallic or multi-metal alloy compounds. Transition metal alloys can offer superior activity directly because of their excellent electrical conductivity and intrinsic polarity. However, most nanoparticles still suffer from inadequate stability and self-aggregation, particularly in harsh electrochemical environments. To address these problems, some researchers prefer to employ carbon materials to preserve the stability of nanoparticles and prevent aggregation or collapse under extreme electrolyte environments or high and unstable overpotentials. In addition, the unique structure of transition metal alloys offers new novel prospects for electrocatalysis. Every metal atom within the alloy provides two potential active sites for the adsorption of oxygen intermediates and may achieve lower reaction energy barriers through different new reaction mechanisms. Two mechanisms described include the modulation mechanism and the bifunctional mechanism, respectively [95].

**Figure 7:**
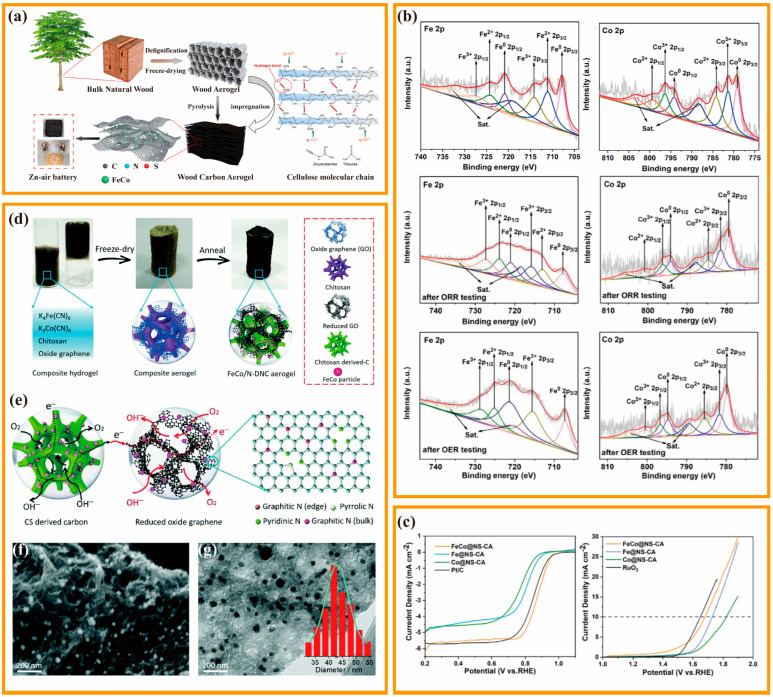
(**a**) Schematic diagram for the synthesis of FeCo@NS−CA. (**b**) XPS results before and after catalyst cycling test. (**c**) ORR and OER curves of FeCo@NS−CA and comparison samples. Copyright 2021 by the American Chemical Society from reference [98]. (**d**) Schematic diagram for the synthesis of FeCo/N−DNC aerogels. (**e**) Schematic diagram of the structural advantages of FeCo/N−DNC aerogels as an efficient bifunctional catalyst. (**f**) SEM and (**g**) TEM images of FeCo/N−DNC. Copyright 2018 by the Royal Society of Chemical from reference [14].

The modulation mechanism involves the electronic regulation of a metal atom by another neighboring metal atom, thereby promoting the intrinsic activity of the previous metal atom, serving as the actual active center [96,97]. Specifically, only one metal atom in the metal alloy acts as the actual active center. It interacts directly with the ORR/OER intermediate, while the other metal atom acts as an environmental atom, modulating the electronic structure without directly adsorbing the intermediate. Pang et al., proposed a convenient delignification method for converting natural balsa wood into layered porous carbon materials, and prepared N, S-doped wood-based CA-supported FeCo alloy (FeCo@NS-CA) and used it as the cathode of the rechargeable ZAB (Figure 7a) [98]. The obtained FeCo@NS-CA with a porous layered structure exhibits an excellent bifunctional electrocatalytic performance and apparently outperformed the single metal catalyst. Moreover, the active sites were determined by comparing the XPS results before and after the catalyst cycling tests (Figure 7b). The results showed that the Fe 2p content was almost unchanged after cycling and the Co 2p content almost disappeared, which means that the main reactive site in the ORR and OER reactions is Fe. Further investigation revealed that Fe_x_-N was the true active site in the ORR reaction, whereas Fe^3+^ was primarily responsible for the OER. Furthermore, the initial voltage of ZAB based on FeCo@NS-CA is close to 1.4 V, and its power density is 140 mW·cm^−2^, showing excellent battery performance. Similarly, Fu et al., prepared a Fe/Co co-doped double network CA (FeCo/N-DNC) catalyst (Figure 7d) [14]. The electrocatalytic performances of FeCo/N-DNC and different samples were compared by electrochemical tests. The ORR catalytic performance of FeCo/N-DNC (E_onset_ = 0.98 V, E_1/2_ = 0.81 V) was greatly enhanced relative to the monometallic sample. The high catalytic activity of FeCo alloy, the coupling effect between FeCo and CA nano-double network structure, and the appropriate 3D network structure together contribute to the excellent electrochemical catalytic performance of the material (Figure 7e). Additionally, the catalytic activity of the two monometallic samples also differed, with Fe/N-DNC (E_onset_ = 0.86 V, E_1/2_ = 0.75 V) outperforming Co/N-DNC (E_onset_ = 0.83 V, E_1/2_ = 0.70 V), indicating that the Co doping was beneficial for improving the conductivity and activating the Fe sites of the catalyst (Figure 7f,g).

**Figure 8:**
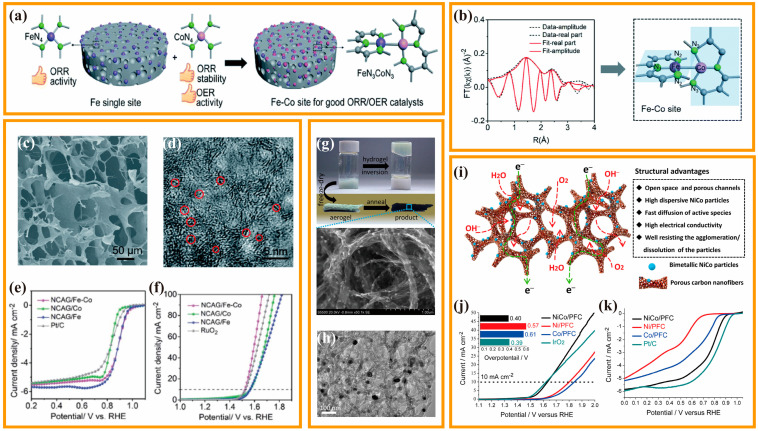
(**a**) Schematic diagram for the synthesis of the NCAG/Fe−Co. (**b**) Fe K-edge EXAFS of NCAG/Fe−Co and the fitting curve and schematic illustration of the structure of the Fe−Co dual metal sites in NCAG/Fe−Co. (**c**) SEM image and (**d**) HRTEM image of the NCAG/Fe−Co aerogel where the red circles marked as metal alloy. (**e**) ORR and (**f**) OER curves of NCAG/Fe−Co. Copyright 2020 by the Royal Society of Chemistry from reference [101]. (**g**) Schematic diagram for the synthesis and SEM images a of the NiCo/PFC aerogels. (**h**) TEM image of the NiCo/PFC aerogels. (**i**) Schematic Illustration of the advantages of the NiCo/PFC Aerogels as electrocatalysts. (**j**) OER and (**k**) ORR curves of the NiCo/PFC aerogels and comparison samples. Copyright 2016 by the American Chemical Society from reference [102].

The bifunctional mechanism mainly involves that two metal atoms adjacent to each other in a metal alloy can catalyze ORR and OER, respectively, thus achieving bifunctional electrocatalytic activity [99,100]. Since ORR and OER have different reaction mechanisms and reaction rates, electrocatalysts with multiple active sites are required. Many studies have also demonstrated the bifunctional electrocatalytic properties of transition metal alloys. Chen et al., developed N-doped CA with Fe-Co bimetallic sites (NCAG/Fe-Co) using the modified gelatin gel as a 3D template and precursor (Figure 8a) [101]. The results from X-ray absorption spectroscopy and other measurements demonstrate that FeN_3_ and CoN_3_ are partially formed on mutually orthogonal planes, with the direct interaction between Fe and Co bonds (Figure 8b–d). Furthermore, it is demonstrated that Co considerably enhances the 4e^−^ ORR selectivity of Fe for bifunctional electrocatalysis by preventing undesirable H_2_O_2_ byproducts. Simultaneously, in contrast to the NCAG/Fe and NCAG/Co samples based on single-atom, the Fe-modulated Co atoms exhibit controlled oxygen intermediate adsorption energy, resulting in faster OER kinetics (Figure 8e,f). Electrochemical tests indicate that NCAG/Fe-Co exhibits exceptional bifunctional electrocatalytic activity, with a mere 0.64 V ORR/OER potential difference at 10 mA·cm^−2^. When applied in a flexible ZAB, the dual-metal NCAG/Fe–Co catalyst also shows a remarkable performance, with a high open-circuit voltage of 1.47 V, a maximum power density of 117 mW·cm^−2^, as well as good rechargeability and flexibility. Apart from Fe and Co, other transition metal atoms within the metal alloy structure have also been observed to exhibit a bifunctional effect, enhancing the intrinsic ORR/OER performances. Fu et al., reported the exceptional performance of NiCo nanoparticles supported on porous fibrous CA (NiCo/PFC) as bifunctional catalysts for ZAB (Figure 8g,h) [102]. Ni/PFC and Co/PFC were also produced as the control samples of monometallic Ni or Co atoms, respectively. Through a comparison of the intrinsic activity of Ni/PFC and Co/PFC, it has been discovered that the Co atoms function as active sites for ORR, whereas the OER electrocatalytic activity can be attributed to the Ni atoms. The presence of Ni-Co dual-metal sites in NiCo/PFC results in not only higher ORR activity (E_onset_ = 0.92 V, E_1/2_ = 0.79 V) than Co/PFC (E_onset_ = 0.85 V, E_1/2_ = 0.68 V), but also higher OER activity (E_10_ = 0.40 V) than Ni/PFC (E_10_ = 0.57 V) (Figure 8i–k). This outcome suggests that the proximity of metal atoms promotes the reaction on both Ni and Co sites, increasing electrocatalytic activity for both ORR and OER processes.

**Table 2:** Summary of various catalysts of catalytic performance.

Catalyst	Electrolyte	ORR and OER Performance			Metal–Air Batteries Performance		Ref
		ORR Performance		OER Performance	Open Circuit Potential (V)	Peak Power Density (mW cm^−2^)	
		E_onset_ (V vs. RHE)	E_1/2_ (V vs. RHE)	E_10_ (mV vs. RHE)			
N-CNFs	0.1 M KOH	−0.23 (V vs. Ag/AgCl)	−0.18 (V vs. Ag/AgCl)				[79]
Pt/C	0.1 M KOH	−0.18 (V vs. Ag/AgCl)	−0.11 (V vs. Ag/AgCl)				
N-CNF	0.1 M KOH	0.83	0.80		1.5		[80]
Pt/C	0.1 M KOH	0.85	0.75		1.5		
BN-CA-900	0.1 MKOH	0.91	0.77				[81]
Pt/C	0.1 M KOH	0.93	0.78				
N_1_S_1_-CNW-900	0.1 M KOH	0.905	0.838				[82]
Pt/C	0.1 M KOH	0.931	0.851				
Fe-N-CA	0.1 M KOH	0.918	0.798				[87]
	0.5 M H_2_SO_4_	0.801	0.592				
Pt/C	0.1 M KOH	0.958	0.833				
	0.5 M H_2_SO_4_	0.921	0.701				
NCA_LR_/Fe	0.1 M KOH	1.05	0.88		1.81	181.1	[88]
Pt/C	0.1 M KOH	0.99	0.84		1.80	175.1	
FeNC-900-8	0.1 M KOH		0.88		1.67	124.9	[89]
Pt/C	0.1 M KOH		0.84		1.50	87.6	
Fe-N-CNTA	0.1 M KOH	0.97	0.88				[90]
Pt/C	0.1 M KOH	0.96	0.86				
Co-SAs/NCNA	0.1 M KOH	0.98	0.86		1.49	206	[91]
Pt/C	0.1 M KOH	0.95	0.84			167	
FeP/Fe_2_O_3_@NPCA	0.1 M KOH	0.95	0.838	464	1.428	130	[92]
Pt/C	0.1 M KOH	0.926	0.842	402	1.3	108	
CoFe_2_O_4_/CNF	0.1 M KOH	−0.09 (V vs. Ag/AgCl)	−0.21 (V vs. Ag/AgCl)	800 (mV vs. Ag/AgCl)			[93]
Pt/C	0.1 M KOH	−0.07 (V vs. Ag/AgCl)	−0.138(V vs. Ag/AgCl)	760 (mV vs. Ag/AgCl)			
HDCA-800	0.1 M KOH	0.94	0.79				[94]
Pt/C	0.1 M KOH	0.98	0.80				
Co_9_S_8_/N,P-APC	0.1 M KOH	0.89	0.78	363			[16]
Pt/C	0.1 M KOH	0.98	0.83	368			
FeCo@NS-CA	0.1 M KOH	0.97	0.85	450	1.40	140	[98]
Pt/C	0.1 M KOH	1.00	0.85	420	1.40	122	
FeCo/N-DNC	0.1 M KOH	0.98	0.81	390	1.50	115	[14]
Pt/C	0.1 M KOH	0.98	0.84	380	1.50	109	
NCAG/Fe-Co	0.1 M KOH	1.04	0.89	293	1.47	117	[101]
Pt/C	0.1 M KOH	0.96	0.83	297	1.36	92	
NiCo/PFC	0.1 M KOH	0.92	0.79	400			[102]
Pt/C	0.1 M KOH	0.94	0.84	390			
NSCA/FeCo	0.1 M KOH	0.97	0.82	335	1.496	132	[103]
Pt/C	0.1 M KOH	0.92	0.821	332	1.496	101.8	
Fe-SA@PNC	0.1 M KOH	0.98	0.87		1.45	149	[104]
Pt/C	0.1 M KOH	0.95	0.83		1.40	119	

## 4. Summary and Outlook

Biomass shows great advantages such as broad sources, low cost, easy access, massive abundance, and being environmentally friendly. Therefore, the synthesis of biomass-based porous CA is an economical and sustainable application method. In recent years, 3D CA has achieved increasing attention due to its large specific surface area, exceptional porosity, low density, superior electrical conductivity, and good biocompatibility, and thus shows great application potential and broad application prospects in the fields of adsorption materials, supercapacitors, and fuel cells. The 3D network structure of biomass-based CA is controllable and easily modified by doping with heteroatoms and metal nanostructure composition. The large specific surface area can supply a large number of active sites for the catalyst, acting as the catalyst carrier for efficient reactions like fuel cells. In addition, CA also has superior electrochemical characteristics, including high electrical conductivity, and structural stability beneficial for fast charge–discharge. This paper mainly discusses the application of biomass-based CA in the field of electrocatalysis.

Catalysts play a key role in electrocatalytic reaction. An excellent electrocatalyst can effectively reduce the reaction overpotential, increase the current density, enhance the electrocatalytic efficiency, and reduce the cost of electrocatalysis. Currently, the development of inexpensive, high-performance non-noble metal electrocatalysts is significantly important for solving the current energy and environmental issues and substituting expensive precious metal catalysts. CA catalyst supports have received extensive attention owing to their tunable pore structure and stable physicochemical properties, and several studies have been carried out on CA-supported non-precious metal and non-metal doped catalysts. Through doping, defect engineering, surface functionalization, and other structural modification of biomass carbon materials, the catalytic performance of biomass-based carbon materials has even exceeded some noble metal catalysts in laboratory tests. Therefore, using biomass-based carbon materials is expected to be a promising approach to solving global energy and environmental challenges. 

Based on the technical review, the following perspectives are pointed out to promote the widespread application and advancement of biomass-derived carbon-based electrocatalysts in the energy field.

(1) Complex process for preparing CAs. Commonly used synthesis processes are supercritical drying and freeze drying, which consume a large amount of energy for the drying process. In addition, the existing technology cannot freely adjust the structure of the holes in a wide range. Also, the poor mechanical properties of CA are limited in industrial production.
(2) At present, research on the modulation of the electronic structure of catalysts by CAs, the regulation of heteroatom doping configuration, the interaction mechanism between CAs and catalysts, and the catalytic mechanism of CA-based catalysts is inadequate. Therefore, it is imperative to conduct a deep and systematic study of the active sites and reaction mechanisms of carbon-based catalysts through theoretical calculations, in situ characterization, and electrochemical testing techniques, and to prepare and optimize the proper catalyst structure under the guidance of basic theories.
(3) The wide variety of biomass leads to certain differences in the structures and compositions among them. Therefore, it is particularly significant to focus on improving the universality of the preparation method of biomass carbon-based catalysts by adjusting the uniformity of catalyst structure and catalytic active sites.
(4) Due to the diversity and compositional complexity of biomass, the specific chemical composition of biomass carbon obtained after the activated carbonization of biomass, including the percentage of heteroatoms, cannot be precisely controlled, making it difficult to ensure the consistency of its performance as an electrode for metal–air batteries.
(5) Multiple steps are required to prepare most biomass-based CA materials such as biomass decomposition and carbonization. For the preparation of air electrodes, powder carbon materials need to be additionally sprayed onto carbon cloth, reducing the energy efficiency. Therefore, the rational maintaining of the original structure of biomass to prepare 3D integrated catalytic electrode materials has significant application prospects.
(6) CA-supported non-noble metal catalysts and non-metal doped CA catalysts are the main directions for the future development of fuel cell catalysts. However, both still face application problems such as low catalytic activity, poor stability, doping technology load, etc. Therefore, further improvements in the performance of these two catalysts are necessary. It is believed that CA-based catalyst technology could be applicable in electrochemical catalysis in the near future.

## Figures and Tables

**Table 1:** Comparison of the characteristics of different types of aerogels.

Aerogel Categories	High Specific Surface Area	Hierarchical Porous Structure	Conducting Skeleton	Self-Supportability	Renewable Resource
Biomass-derived carbon aerogels	Yes	Yes	Yes	Yes	Yes
Oxide aerogels (e.g., Silica aerogel)	Yes	Yes	No	Yes	No
Pure metallic aerogels	Yes	Yes	Yes	Yes	No

## Data Availability

The authors declare that all data supporting the findings of this study are available within the article.
